# Good Rates From Bad Coordinates: The Exponential Average Time-dependent Rate Approach

**DOI:** 10.1021/acs.jctc.4c00425

**Published:** 2024-07-02

**Authors:** Nicodemo Mazzaferro, Subarna Sasmal, Pilar Cossio, Glen M. Hocky

**Affiliations:** † Department of Chemistry, New York University, New York, New York 10003, United States; ‡ Center for Computational Mathematics, Flatiron Institute, New York, New York 10010, United States; § Center for Computational Biology, Flatiron Institute, New York, New York 10010, United States; ∥ Simons Center for Computational Physical Chemistry, New York University, New York, New York 10003, United States

## Abstract

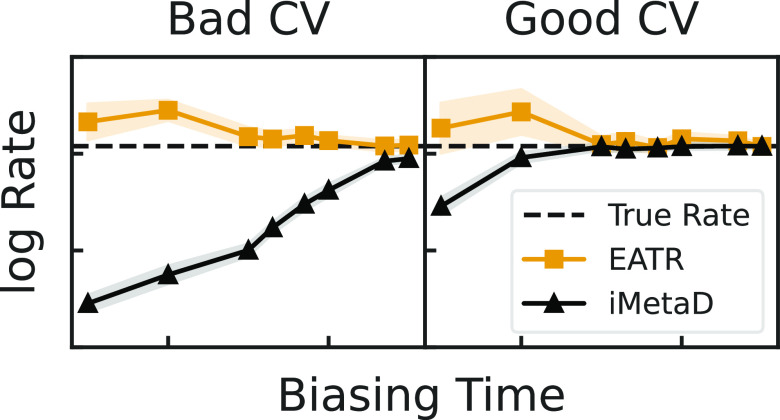

Our ability to calculate rate constants of biochemical processes using molecular dynamics simulations is severely limited by the fact that the time scales for reactions, or changes in conformational state, scale exponentially with the relevant free-energy barrier heights. In this work, we improve upon a recently proposed rate estimator that allows us to predict transition times with molecular dynamics simulations biased to rapidly explore one or several collective variables (CVs). This approach relies on the idea that not all bias goes into promoting transitions, and along with the rate, it estimates a concomitant scale factor for the bias termed the “CV biasing efficiency” γ. First, we demonstrate mathematically that our new formulation allows us to derive the commonly used Infrequent Metadynamics (iMetaD) estimator when using a perfect CV, where γ = 1. After testing it on a model potential, we then study the unfolding behavior of a previously well characterized coarse-grained protein, which is sufficiently complex that we can choose many different CVs to bias, but which is sufficiently simple that we are able to compute the unbiased rate directly. For this system, we demonstrate that predictions from our new Exponential Average Time-Dependent Rate (EATR) estimator converge to the true rate constant more rapidly as a function of bias deposition time than does the previous iMetaD approach, even for bias deposition times that are short. We also show that the γ parameter can serve as a good metric for assessing the quality of the biasing coordinate. We demonstrate that these results hold when applying the methods to an atomistic protein folding example. Finally, we demonstrate that our approach works when combining multiple less-than-optimal bias coordinates, and adapt our method to the related “OPES flooding” approach. Overall, our time-dependent rate approach offers a powerful framework for predicting rate constants from biased simulations.

## 1 Introduction

A major challenge in biomolecular simulation is to be able to accurately assess the transition rate constant (inverse of the mean residence time in a state) of complex processes, including conformational transitions and the binding/unbinding of macromolecules and their ligands. Processes of interest often involve rare events, where the system spends a large amount of time in a metastable state and rarely transitions to another relevant one, so the transition path time is typically orders of magnitude shorter than the time spent in either state.^1^ Because of this, extracting rates of such processes directly from unbiased simulation is out of reach for all but the simplest of systems.

Numerous methodologies have been developed to accelerate rare conformational transitions, with the primary purpose being to compute ensemble-averaged observables.^2,3^ A major subclass of such methods operate by adding an additional biasing potential to the system’s Hamiltonian, usually in terms of a small set of collective variables (CVs) which are believed or determined to be good descriptors of states of interest, or the path between them.^2,3^ Common examples of such methods include Umbrella Sampling, Adaptive Bias Force, Metadynamics (MetaD), Variationally Enhanced Sampling, and On-the-fly Probability Enhanced Sampling (OPES), among others.^4−12^ All of these methods pay the price of distorting the system’s dynamics to obtain a much more rapid estimate of the underlying free-energy landscape as a function of the CVs.

Most methods that tackle the problem of computing rates of rare transitions seek to generate a set of unbiased trajectories, either through combining direct sampling of many short trajectories from different starting points^13,14^ as done in Markov State Modeling, through Monte Carlo in trajectory space as in Transition Path Sampling,^15^ or by generating trajectories that progress in a particular coordinate as in Forward Flux Sampling,^16^ Steered Transition Path Sampling,^17^ Weighted Ensemble,^18^ Transition Interface Sampling,^19^ and Milestoning.^20^ However, these methods are computationally expensive, and some scale poorly with system size, making them challenging to apply for the complex biophysical problems we are interested in studying, such as finding the time scale for protein–drug unbinding,^21^ for the RBD opening of the SARS-CoV-2 Spike protein,^22^ or for the unbinding of cytoskeletal adhesion proteins under force.^23−25^

As such, we are interested in approaches that build on CV biasing methods, which have been used to probe conformational transitions with sufficient computational efficiency even for relatively complex biological assemblies. The challenge already mentioned is that these methods alter the dynamics, which prevents any obvious solutions to inferring the unbiased time scales of events. However, starting with the Hyperdynamics method of Voter, it was shown that the first passage time of rare events could be approximately predicted using biased simulations if bias is not applied during the actual crossing through the transition state, by formulating an ansatz for how time is accelerated.^26,27^ This approach was originally developed using a time-independent potential defined on the whole system of interest, but later in the Infrequent Metadynamics (iMetaD) approach the same ideas were extended to CV biasing. MetaD^8^ works by updating an external bias with a Gaussian centered at the current position in CV space every Δ time steps (see Section 5.2 for details). iMetaD solves the problem of not biasing the transition over the barrier by only rarely updating the bias potential, such that it is unlikely to add bias on a high barrier during a fast crossing. iMetaD also introduces an additional approximation since the system is experiencing a time-dependent bias rather than a static one. The difficulty of avoiding adding bias during barrier crossings can also be mitigated by MetaD variants that only add bias within a region or up to a certain energy level,^28,29^ which is now particularly easy to implement in the OPES variant of MetaD.^12,30^ iMetaD and similar approaches have now been used and benchmarked for many different problems, especially for protein–ligand unbinding problems,^31^ as reviewed in ref (32).

To extract the transition rate, these methods assume that a “good” CV is used, and validate the rate estimates using a Kolmogorov–Smirnov (KS) test between the empirical and theoretical survival distributions. Unfortunately, for large and complex transitions, the CV that is used may be poor because finding a good CV is challenging. Moreover, CV quality indicators, such as the committor,^33^ are expensive or intractable to compute. The Kramers time-dependent rate (KTR) method^34^ was recently developed to extract transition rates from biased simulations, such as those used for iMetaD, but with much less sensitivity to CV choice. It introduced a new parameter γ, called the CV biasing efficiency, that scales the effect of the added potential. In that work, it was shown that γ had a lower value for a poor CV in a simple 2D double-well potential, and as such it was assumed to relate to the CV quality.^34^ However, this has not been systematically demonstrated, and KTR has not been benchmarked on a problem where many CVs could be tested. Moreover, a direct connection between the KTR and iMetaD estimators has not been established.

In this work, we introduce a more general framework for computing rates from time-dependent biasing protocols, which allows us to treat the iMetaD and KTR estimators on the same footing. We then use this framework to propose a revision to KTR termed the Exponential Average Time-dependent Rate (EATR) method that bridges the two approaches. The EATR approach is shown to give the correct Kramers’ rate when γ = 1 for an idealized 1D potential. Then, we use a Go̅-protein system as a model to show how the prediction of rates depends on the choice of bias coordinate, and compare EATR’s results to the true intrinsic rate. Importantly, we find that γ correlates with the intuition of CV quality. We find that for the poor biasing coordinates, the original KTR and EATR results are comparable and they enable an accurate recovery of the unbiased rates. Surprisingly, this is often true even in the frequent-biasing regime. These same overall conclusions hold when applied to the folding of the small peptide chignolin, using biased trajectories along three CVs provided by the authors of ref (35).

The paper is organized as follows. First, we present a general theory for rate calculations from time-dependent biased simulations. We relate it to iMetaD and KTR, and then formulate the EATR approach. Then, we show results for a 1D overdamped Langevin dynamics simulation, and for the unfolding process of two proteins for which we can easily compare by biasing different CVs. We also adapt our approach to be used with OPES rather than a MetaD biasing protocol. We show that the method can be extended beyond one biasing coordinate, presenting accurate results on protein G unfolding when biasing two CVs simultaneously. We end with conclusions and future perspectives of the work.

## 2 Theory

### 2.1 Transition Rate for Rare Events

The rare event problem constitutes the stochastic crossing of a single free-energy barrier, where typically the waiting time to cross the barrier is much longer than the transition time over it. For a high barrier, the survival function *S*(*t*), which is the probability of a transition not occurring before time *t*, is given by an exponential distribution characterized by a single transition rate constant *k*_0_
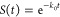
1

Note that this survival probability is related to the probability of a transition occurring at time *t* via
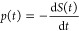
2and it is also related to the cumulative distribution function (CDF), the probability that a transition occurred by time *t*
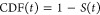
3

For Brownian dynamics, Kramers’ rate theory^36−39^ can be used with several approximations to calculate the barrier crossing rate,⊥*k*_0_, from the bottom of a well on a potential surface *U*(*x*) containing a single high barrier
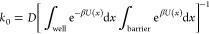
4where *D* is the diffusion coefficient.

However, for most systems of interest, the diffusion coefficient and underlying potential (or free-energy landscape) are not known and, therefore, one cannot directly use eq 4 to estimate the rate. Instead, the transition rate constant can be calculated using the survival function and a set of simulations *i* = 1, ..., *N* where *M* ≤ *N* have crossed the barrier and *N* – *M* have not. Let *t*_*i*_ be the time the *i*-th simulation crossed the barrier, and *t*_*i*_ = *T*_*i*_ the total simulation time for simulations *i* = *M* + 1, ..., *N*. For right censored transition times, the likelihood is given by
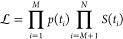
5which is the product of the probabilities of the transitions occurring at times  and the probabilities of not transitioning before the times  for *N* – *M* simulations.^40^

An estimate for the transition rate constant can be obtained by substituting eq 1 and eq 2 into eq 5 and maximizing the logarithm of the likelihood with respect to *k*_0_
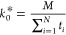
6

Note that the summation in the denominator takes into account the simulations that did not transition. When all simulations have crossed the barrier, eq 6 reduces to the inverse of the average barrier-crossing time,  where ⟨·⟩ denotes the average over the simulations.

The transition rate can also be calculated by fitting the CDF (eq 3). To do so for the same set of simulations *i* = 1, ..., *N*, we construct an empirical CDF which is the number of simulations that have transitioned before *t*_*i*_ over the total number of simulations. The theoretical CDF can then be fit to the empirical CDF with a least-squares method by optimizing *k*_0_.

### 2.2 Expression for the General Time-dependent Rate

For time-dependent biased simulations, such as a MetaD simulation, the transition rate is no longer a constant, and hence we would like to formulate a general expression for the survival function in the case of a time varying potential (similar to a situation considered by Zwanzig in ref (41).). Without loss of generality, we can write
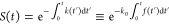
7which can be used in eq 3 for fitting to an empirical CDF. Here, we introduced a time-dependent rate constant *k*(*t*), and then re-express *k*(*t*) as the unbiased *k*_0_ scaled by a function of time
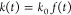
8

Because *S*(*t*) = e^log(*S*(*t*))^, this is equivalent to defining , and we can do this because we expect log(*S*(*t*)) to be differentiable at *t* > 0 for a physically realizable process.

Substituting eq 7 into eq 5 results in a general likelihood given by
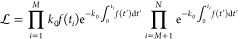
9which can be simplified by taking its logarithm
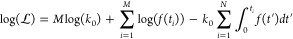
10

Similar to the unbiased case, we can maximize this expression with respect to *k*_0_, to obtain *k*_0_^*^ as an estimator for the true rate constant
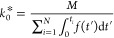
11

Maximization of the log likelihood function answers the question: what *k*_0_ best describes the observed biased survival times assuming the particular form of the time dependent rate constant given by eq 8.

### 2.3 Relation to iMetaD

In the hyperdynamics method,^26,27^ the rate from transition state theory is scaled by the acceleration factor  (note that here we use the subscript *X* to denote a configurational average and unlabeled brackets to represent an average over separate trajectories) where *V*(*x*) is a fixed bias function added to the system’s Hamiltonian as a function of the system’s full coordinates. This α arises by considering the average effect in many individual trajectories whose time is dilated by a factor , where *V*_*i*_(*t*) is the bias experienced by the system during simulation *i* at time *t*. Hyperdynamics then corresponds to a rate scaling function of the form
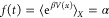
12and inserting this in eq 7 results in the survival probability
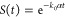
13

Using this expression and assuming all simulations transitioned (*M* = *N*), the likelihood maximization (LM) gives
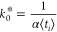
14

In iMetaD, the form of the bias is also changing in time along with the configuration of the system in a history-dependent manner. Therefore, in iMetaD an acceleration factor for each simulation is approximated as a time average over that simulation instead of calculating it as a configurational average, . In the Supporting Information Section S1, we show that using *f*(*t*) = e^β*V_i_*(*t*)^, we recover the standard rescaling formula used in iMetaD
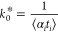
15

We note that this result is derived using the LM approach for the case where all simulations have transitioned.

In ref (42), it was shown that directly fitting the theoretical CDF (obtained from eq 13) is less sensitive to outliers in the tail of the distribution. The KS test can be used to assess whether the transition distribution is well-described by the theoretical CDF (see Section 5.6). Recently, it was also suggested that the short time information from the CDF can be fit to get a more robust estimate of the rate.^35^ We note that the results from LM and CDF fitting need not coincide, as we will describe below.

### 2.4 Kramers Time-dependent Rate and the CV Biasing Efficiency

Most of the transition-rate methods for biased simulations, such as those described above, are formulated assuming that it is possible to apply the bias along a perfect CV, where all added bias accelerates the barrier crossing event. However, for large biomolecular systems, choosing a priori a perfect CV for accelerating transitions to another targeted state is almost impossible. In practice, the bias is applied along nonideal CVs, which insert bias along useless directions that are not aligned with the transition path.

To overcome this issue, the KTR theory^34^ introduces a parameter, γ ∈ [0, 1], to account for the efficiency of the biased CVs. In addition to the unbiased rate, γ will also be estimated from the simulation transition times, and it will inform about the quality of the CV with γ → 0 reflecting poor CVs and γ ∼ 1 good ones. In the KTR approach as previously implemented, the efficiency of CVs is accounted for by defining the scaling function as
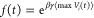
16where ⟨max *V*_*i*_(*t*)⟩ is the maximum bias applied at any point up to time *t* in simulation *i*, averaged over all simulations (denoted *V*_*MB*_(*t*) in ref (34).). This form of treating the biasing potential was inspired by rate-calculation methods developed for force-spectroscopy,^43,44^ where the barrier is reduced due to the external force, and therefore, by using ⟨max *V*_*i*_(*t*)⟩, it was assumed that the bias only affects the barrier height. Inserting eq 16 into eq 7 gives
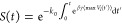
17which can be used directly in a CDF fit. Substituting this expression into the log-likelihood from eq 10, and maximizing it with respect to *k*_0_ results in a γ-dependent expression for the unbiased rate constant
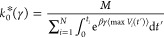
18

To obtain the maximum likelihood estimate for both γ and *k*_0_, eq 18 is substituted back into the log-likelihood function and it is maximized numerically with respect to γ.

### 2.5 Exponential Average Time-dependent Rate (EATR)

While rate constants computed by the KTR approach are accurate (as shown in ref (34) and in the following sections), we show below in Section 3.1 that it has the undesirable property that it does not agree with the iMetaD estimator when γ = 1, whereas we expect the iMetaD estimator to be correct for an ideal coordinate with a very high barrier and slow deposition time. The reason for this discrepancy is the way in which the average effect of the bias is defined—for iMetaD  is averaged, whereas for KTR, the maximum of the biasing potential is averaged.

To unify the two theories, we propose the following modification to the KTR method, which will have the desired property of producing the same rates as iMetaD in the case where γ = 1. To do so, we introduce the scaling function
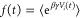
19

This gives the survival probability
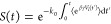
20

Substituting this expression in eq 10 results in a log-likelihood of the form
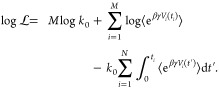
21

In the case where all simulations transition (*M* = *N*), the optimal unbiased *k*_0_ as a function of γ is given by
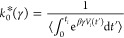
22where we have benefited from the idempotence of averages to rewrite the average of an average as a single average. Observing that when γ = 1, the term within brackets in the denominator is equivalent to *t*_*i*_α_*i*_, this estimator is then identical to the standard iMetaD estimator in eq 15. Similarly, as with the KTR, we can now substitute eq 22 into the log-likelihood and numerically maximize it with respect to γ to obtain estimates for both the unbiased rate constant and the efficiency of CVs. Incidentally, it might seem from the final step of this derivation that taking the average in eq 19 was redundant; however, in Section S2 we show that without doing this average, the log-likelihood cannot be maximized with respect to γ.

Importantly, we note that eq 20 also provides us the option to numerically fit the biased empirical CDF to find the best values of *k*_0_ and γ. As initial guesses, we use the LM estimates for *k*_0_ and γ, and optimize using the Levenberg–Marquardt algorithm implemented in the SciPy Python package^45,46^ to fit the empirical CDF to the theoretical CDF obtained from eq 20. The same can be done for the KTR method using the theoretical CDF from eq 17. We note that these optimization procedures are stable for time-dependent biases. In Section 3.2.2 and the Supporting Information, we explore their combination with OPES flooding^30^ that effectively has a time independent bias.

## 3 Results and Discussion

### 3.1 Benchmarking on a 1D Potential

**Figure 1:**
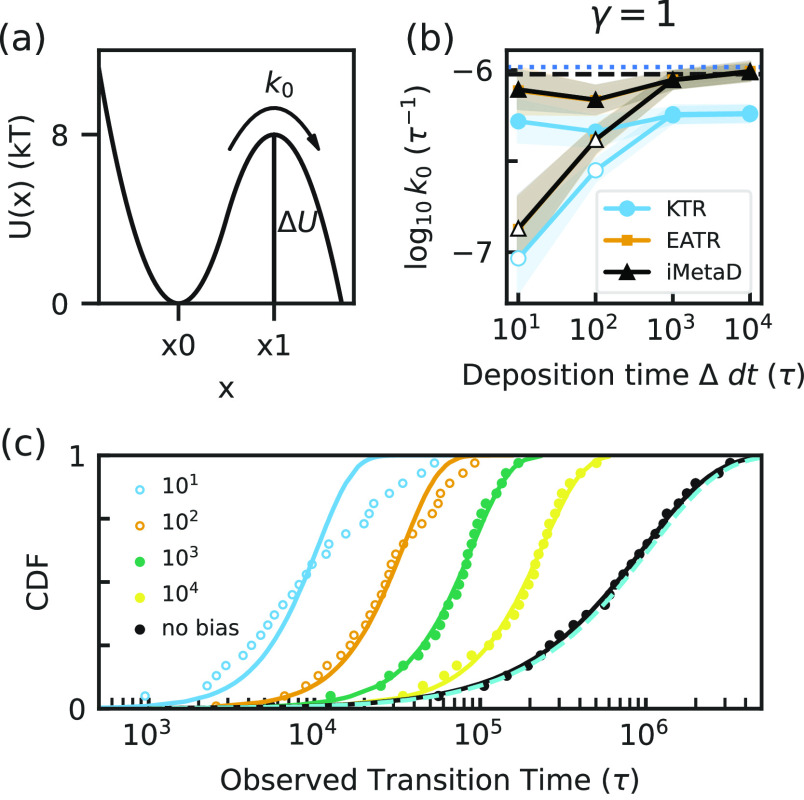
(a) The potential energy profile of the 1D matched-harmonic potential from eq 23. (b) The unbiased rate constant from Kramers’ rate theory and from unbiased simulations are shown as the dashed black line and the dotted blue line, respectively. We compare these with predictions from iMetaD, and from the KTR and EATR methods by asserting γ = 1 as a function of the bias-deposition time (Δd*t*). Maximum likelihood estimates are represented by open symbols while CDF-fit estimates are filled symbols. Error bars are from a bootstrap analysis as described in Section 5.5. (c) The empirical CDFs of observed transition times over the barrier are shown with their EATR-CDF fits; different bias deposition times are indicated in the caption with curves with fastest to slowest biasing appearing from left to right. Fits that fail the KS test are represented with open symbols. The unbiased empirical CDF (black points) is shown together with the Poisson-process distribution fit (black line) and the predicted distribution using the Kramers’ analytical expression, eq 24 (cyan dashed line).

The rate methods were tested first on the one-dimensional matched-harmonic potential illustrated in Figure 1a given by
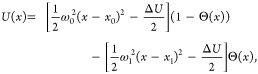
23where the subscript 0 corresponds to the well and the subscript 1 corresponds to the barrier, Θ(*x*) is the Heaviside step function, and , which is needed to make the potential continuous. Full simulation details for this model are given in Section 5.1.

To estimate the unbiased rate constant, many Langevin dynamics simulations were performed on the potential from eq 23, starting from the bottom of the well (Figure 1a). The first barrier-crossing time for each simulation was recorded, and the empirical CDF was calculated as described in Section 2. The unbiased rate constant was extracted by fitting the distribution to the expected Poisson process. The 2-sample KS test was performed to assess whether this transition is accurately described by a Poisson process. The *p*-value for the KS statistic is 0.97, demonstrating that the transition times are likely Poisson-distributed data. This yielded a log_10_ value of −5.98 ± 0.04 where rate constants are in units of τ^–1^, with τ as the time unit, and the error is the standard deviation obtained from bootstrap analysis^47^ (see Section 5.5). The log_10_ estimate calculated with Kramers’ theory using eq 4 is −6.02, which agrees with the empirical rate constant within error.

We then performed well-tempered metadynamics (WT-MetaD) simulations (see Section 5.2) for this system to predict the rates using iMetaD, KTR, and EATR using different bias-deposition times Δd*t* with d*t* the MD time step (varying from 10 to 10,000 τ, which corresponds to fractions of the mean-first passage time varying from 10^–6^ to 10^–2^). In Figure 1, we compare the methods for both the LM and CDF-fit for the situation where γ = 1 is enforced, because (*i*) this allows us to only assess the quality of the different time-dependent rate metrics, and (*ii*) for a 1D potential, all bias should go into promoting the transition as there are no orthogonal degrees of freedom; results obtained from fitting both *k*_0_ and γ are shown in Figure S1. We find that the original KTR method does not give a rate consistent with the empirical unbiased rate when γ = 1. On the other hand, we find that both the iMetaD and EATR methods are consistent with the expected values for the rate. Moreover, in agreement with ref (42), we find that fitting the CDF provides more accurate rate estimates than LM at small Δ for all three methods, with the discrepancy between the two fits negligible for large Δ. The rate estimates for each fitting procedure improve as Δ increases, which is consistent with the principles of iMetaD. This is also consistent with the results of the KS test. The 2-sample KS test was performed on iMetaD and the 1-sample test was performed for KTR and EATR as explained in Section 5.6. The KS tests failed for the CDF fits at Δd*t* = 10^1^ τ and 10^2^ τ and passed for the CDF fits at Δd*t* = 10^3^ τ and 10^4^ τ. These KS test failures are shown for EATR in Figure 1c as open circles. Given that the rate estimates are more accurate for fitting the CDF (even when the KS test fails), we report results from the CDF-fitting procedure below.

### 3.2 Protein G Unfolding

#### 3.2.1 Application of KTR and EATR under WT-MetaD Bias

We now focus on a more complex system, the unfolding of the B1 domain of protein G using a Go̅-like potential and MD simulations. This system has the advantage that it is possible to obtain an unbiased estimate of the unfolding rate, while having a rich unfolding landscape complexity, and many possible choices of CVs to characterize the transition.^48^ Below, we will evaluate the quality of several good and bad CVs for predicting rates. For this study, we first considered the fraction of native contacts *Q* and distance between the ends of the protein (*R*_ee_) which were shown to be a good and bad coordinate, respectively, for characterizing the folding of this protein in ref (48). In addition to these two CVs, we will consider the radius of gyration (*R*_g_), the root-mean-squared-deviation from the native state (RMSD), and a recently developed linear-discriminant analysis coordinate maximally separating states as defined through a clustering analysis^49^ (LD1, see Section 5.4.2, Figure S2).

**Figure 2:**
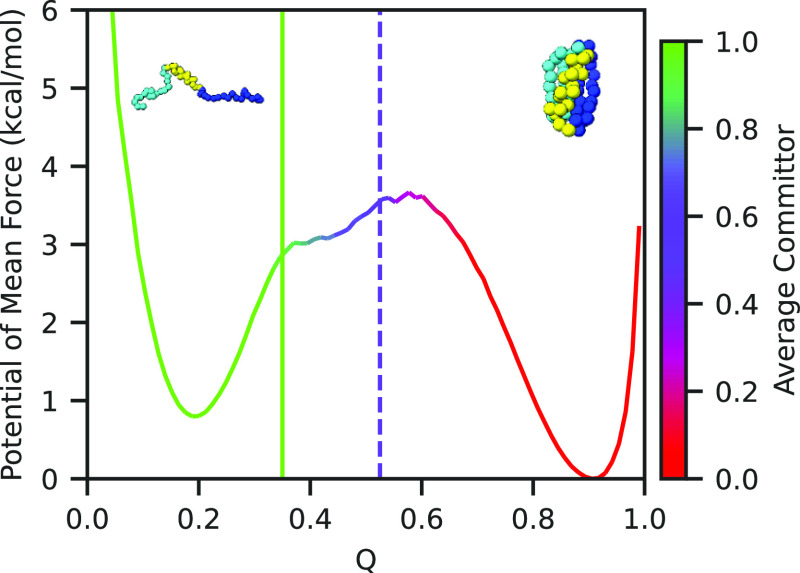
Potential of mean force along the fraction of native contacts *Q* for the Go̅-like model of the B1 domain of protein G colored according to the average committor function. The value of *Q* where the average committor is 0.5 is marked with the dashed line, while the critical value for unfolding *Q* = 0.35 is marked with the solid line.

We first performed a 120 μs-long unbiased simulation to study the system’s behavior. For this unbiased trajectory, we computed the potential of mean force (PMF) along each CV by taking the negative log of the histogram of observed CV values (eq 27). The PMF for *Q* is shown in Figure 2, and along all CVs in Figure S3, revealing a range of potential profiles and apparent barrier heights. Although the PMFs of each CV exhibit two wells, we know that *R*_ee_ is a poor CV for characterizing unfolding because the unfolded ensemble contains configurations with small values of *R*_ee_ comparable to the folded state, resulting in an unusually shaped basin at small values of the CV.

In contrast, we expect *Q* to be a good CV for unfolding,^50^ and so we used *Q* to define when our system has transitioned out of the folded state by using the average committor in the unbiased simulation.^33,51,52^ To do so, we computed for every frame whether the simulation next reached the metastable free-energy minimum on the left (unfolded) before reaching the global minimum on the right (folded), and computed the average by binning these values as a function of the corresponding value of *Q* in that frame. Based on this result, shown in Figure 2, we defined the unfolded region to be where *Q* < 0.35 because the average committor to the left of this point is effectively 1.0. To estimate the unbiased rate, we ran 200 unbiased simulations of the protein with randomized initial velocities, and stopped these simulations when *Q* dropped below *Q* = 0.35 using the COMMITTOR function of PLUMED.^53^ The residence time for the folded state was recorded for each simulation. The unbiased rate constant was determined to be 1.4 ± 0.1 μs^–1^ using the CDF-fitting procedure previously described. The KS test using a Poisson distribution passed with *p* = 0.65, demonstrating a good fit.

**Figure 3:**
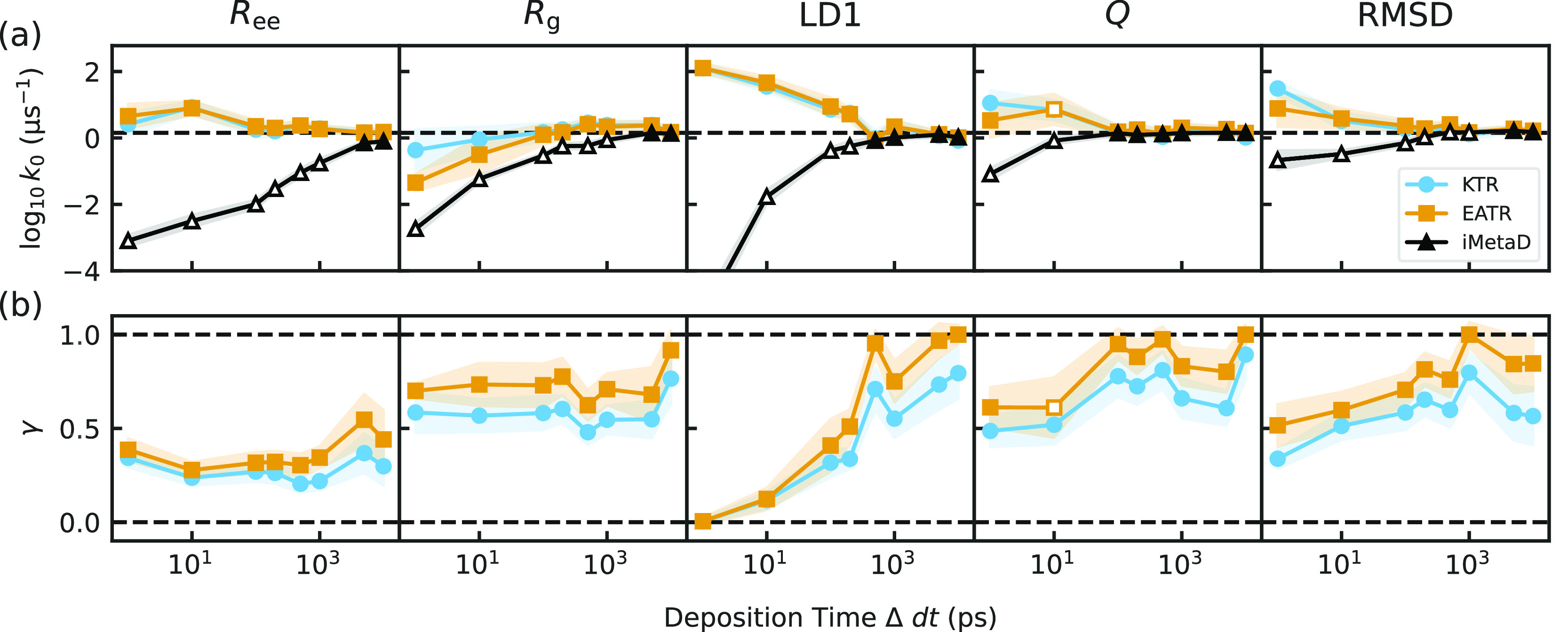
(a) The rate constants obtained from fitting the CDF for iMetaD (black), KTR (blue), and EATR (orange) for each CV at various deposition times (Δd*t*). The horizontal dashed line represents the empirical rate obtained from unbiased simulations. Open shapes indicate where the KS test failed. (b) γ values obtained for the KTR and EATR methods. Horizontal dashed lines represent the bounds placed on γ. In both panels, the error bars are computed from a bootstrap analysis as described in Section 5.5.

We then performed 100 biased simulations for each CV at various bias-deposition times to determine how sensitive each method was to biasing speed. The recovered rate constants from the methods are shown in Figure 3a for each of the biased CVs as a function of the bias-deposition time Δd*t*. Bias deposition times varied from 1 ps to 10 ns, corresponding to fractions of the mean first passage time ranging from ∼10^–6^ to ∼10^–2^ as in the case of the simple potential in the previous section. As expected, longer hill deposition times are observed to generally increase the accuracy of all rate calculations. However, for intermediate to fast-deposition times, KTR and EATR predict unbiased rate constants closer to the true rate than does iMetaD, especially for the three CVs shown on the left of Figure 3a. We also performed a similar study using untempered MetaD and find that, similarly, all methods work well, with KTR and EATR slightly out-performing iMetaD in the fast biasing regime (Figure S4).

The KTR and EATR methods also give a measure of the CV biasing efficiency γ, which is shown in Figure 3b. We find that *Q* and RMSD typically give higher values of γ than the end-to-end distance (*R*_ee_) and the radius of gyration (*R*_g_). This coincides with the physical intuition of protein unfolding CVs, where the number of contacts and the similarity to the folded structure should be most relevant. This also agrees with ref (50), which found that *Q* is a good CV for this system. The CV obtained from linear discriminant analysis (LD1, ref (49).) appears to have a large value of γ for slow biasing and a small value of γ for fast biasing. A similar trend appears for all CVs tested, but this is most prominent in LD1, and we are still investigating the reason γ for LD1 is so much more sensitive than the other CVs here, while still serving as a very good CV for distinguishing folded and unfolded states (as proposed in our previous study^49^). We note that the discrepancy between iMetaD and the true rate constant is most pronounced when KTR or EATR predict lower values of γ.

**Figure 4:**
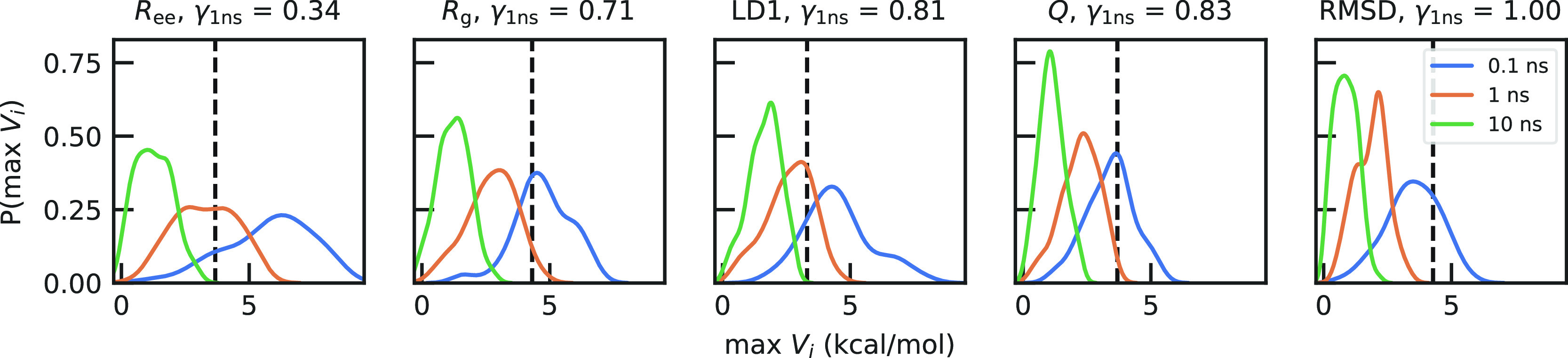
Histograms of the maximum bias deposited for the WT-MetaD simulations for the five CVs and different bias-depositions times. As the biasing efficiency γ increases, there is a decrease in the amount of bias needed for transition.

Our intuitive expectation is that a bad CV would require significant amounts of extra bias to be deposited before the system can overcome the apparent barrier in the FES for that CV, necessitating a low value of γ to compensate in our rate calculation. To check this, we computed the histogram of the maximum bias across the different simulations at different deposition rates (Figure 4). CVs with high γ and slow deposition have maximum bias that do not exceed the apparent barrier, while fast biasing and poor CVs require a substantial amount of extra energy to be injected into the system to effect transitions. Accordingly, if we look at the average bias as a function of position along a poor and good CV (*R*_ee_ and *Q*, respectively, in Figure S5), we find that the amount of bias applied near the transition state is much larger for *R*_ee_ than *Q*. Interestingly, even if some amount of bias is added within the transition region, EATR and KTR are still able to recover the true rate for most cases. We note that we use max *V*_*i*_ in Figure 4 because it is a direct ingredient in the KTR method. Another interesting quantity to compute here would be the amount of nonequilibrium work performed by the MetaD bias, which has been recently exploited in another estimator of rates from time-dependent biased simulations.^54^

For all these CVs, the KTR and EATR methods performed comparably well and consistently performed better than the iMetaD method. Interestingly, the KS test seems not to be as sensitive for KTR and EATR as it was in iMetaD. Fitting the CDF for iMetaD results in failed KS tests even where the error in the rate is small, but KTR never failed the KS test for these CVs and EATR only failed for one condition. This may be an effect of introducing γ as an additional fitting parameter, so it is possible to get fits closer to the empirical CDF with worse rate estimates. In Figure S6, we show that the introduction of γ allows us to make good fits to the CDF; indeed, many pairs of (*k*_0_, γ) can be used to fit these data; however, we note that the resulting predicted rate constants are still quite close to the most confident prediction, so this small amount of flexibility is not a problem here in practice.

#### 3.2.2 Adapting the Approach to OPES Flooding

As mentioned in the introduction, the MetaD-like method OPES offers a promising alternative approach to computing rate constants via the estimator given in eq 15. That is because OPES has a parameter specifying a maximum amount of bias to add, allowing the method to be kept below any apparent barriers to the reaction of interest, and it can be adapted to not include bias outside a prespecified region of CV space.^12,30^ A brief technical description of the OPES-MetaD biasing procedure is given in Section 5.3. Computing rates in this fashion (referred to as OPES flooding by ref (30)) satisfies the assumptions required to derive the iMetaD rate estimator without using the infrequent biasing in iMetaD. The OPES flooding bias rapidly converges to its final value, leading in principle to faster observations of the rare events.^30,32^ We expect that this approach should work better than traditional iMetaD for high dimensional systems such as conformational changes in large biomolecular systems.

**Figure 5:**
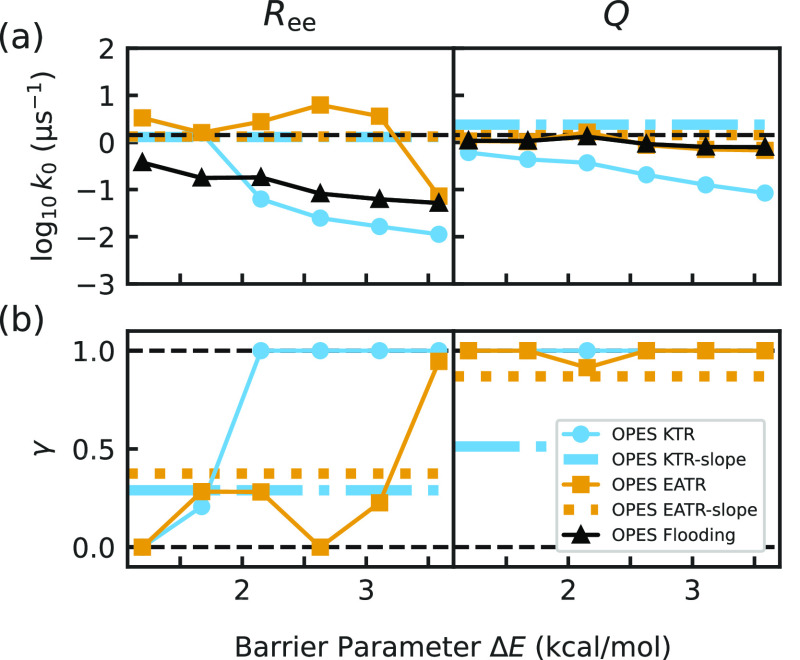
(a) The rate constant estimates obtained from applying the iMetaD, KTR, and EATR methods to OPES simulations when biasing *R*_ee_ (left) or *Q* (right), varying the OPES barrier parameter. (b) The values of γ obtained from these KTR and EATR fits. Colored horizontal dashed lines in both panels represent the more accurate estimates obtained from fitting the observed OPES unfolding rate constant and γ as described in Section S9, denoted OPES-EATR-slope or OPES-KTR-slope, using different OPES barrier simulations; see Figure S7.

In principle, our time dependent rate framework should apply to OPES flooding without modification. We performed six sets of 100 OPES simulations each for the *R*_ee_ and *Q* CVs of the protein G model to compare the different methods. Each set of simulations used a different barrier parameter Δ*E*, ranging between 1 and 4 kcal/mol, with resulting rates shown in Figure 5a. OPES flooding performs comparably to standard iMetaD for both CVs (Figures 5a, S7). Surprisingly, we found that the rapid convergence of the bias function results in a log-likelihood for both KTR and EATR which is insensitive to γ, making it very difficult to maximize; a discussion on why this is the case is given in Section S9. Attempting to fit both in practice often causes instabilities with γ tending toward the extreme values 0 or 1 (Figure 5b), and leading to the inaccurate rate constant estimates shown in Figure 5a. Here, we found that EATR performed better than the standard estimator only in some cases and KTR consistently performed worse. Although we could not find stable solutions for both γ and *k*_0_ by optimizing the likelihood, we discovered that we could fit γ and *k*_0_ by determining how the observed unfolding rate constant scaled with the average of a bias measure as described in Section S9 and Figure S7. This “OPES-EATR-slope” approach results in rate constant predictions accurate to within 5% for both CVs (Figure 5a), but it is an approximation and requires computing and combining the results from simulations biased using different OPES flooding barriers.

#### 3.2.3 Simultaneous Biasing of Two Collective Variables

MD studies involving large biomolecules or molecular assemblies will typically have many slow degrees of freedom characterizing transitions between important states, and hence we expect the need to use multiple CVs to bias the system in order to promote transitions in a reasonably short amount of simulation time. We wanted to assess whether KTR and EATR still work for this case, despite the fact that the role of γ in characterizing CV quality is less direct. To do so, we performed WT-MetaD simulations while simultaneously biasing the end-to-end distance *R*_ee_ and the radius of gyration *R*_g_, and summarize the results in Section S10 and Figure S8. Overall, both KTR and EATR recovered the rate equally well apart from EATR for the fastest bias-deposition time, where the KS test failed. For intermediate values of bias deposition time, the value of γ extracted is higher than that of either CV alone, which could be connected to the fact that biasing multiple CVs simultaneously increases the efficiency of the biasing to produce transitions, and this is something we will investigate more rigorously in the future.

### 3.3 Chignolin Miniprotein Unfolding

**Figure 6:**
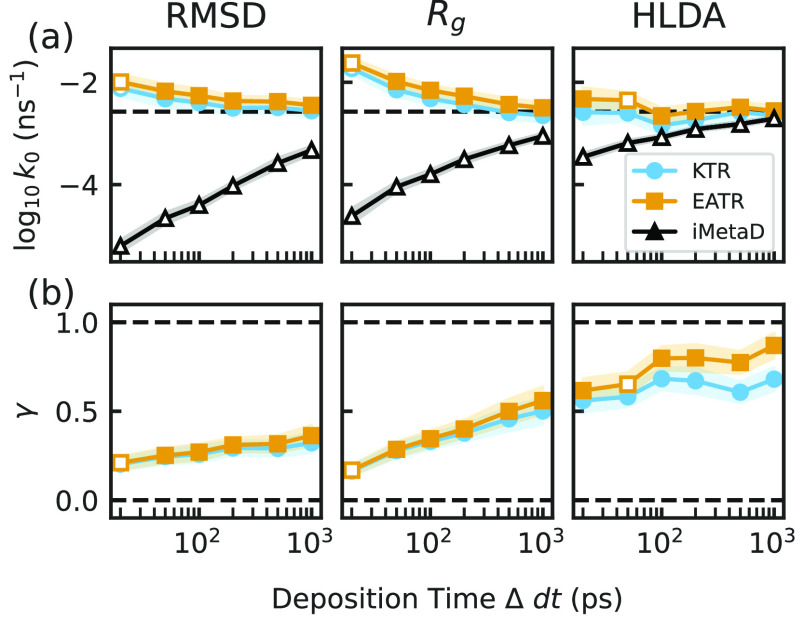
(a) The unfolding rate constants obtained from fitting the CDF for iMetaD (black), KTR (blue), and EATR (orange) at various deposition times for each of the biased CVs of all-atom simulations for the chignolin miniprotein from ref (35). (b) The values of γ for each CV for each deposition time. Open shapes indicate where the KS test failed. The error bars were obtained by bootstrapping with subsamples of 200 simulations out of the 1000 in each set.

To ensure that our method is robust for more complicated atomistic systems, we applied the KTR and EATR methods to the chignolin miniprotein data presented in ref (35). The CVs which were biased in that paper were the C-alpha RMSD to the folded state, the radius of gyration, and a CV obtained from harmonic linear discriminant analysis (HLDA), and for each CV and bias deposition time, 1000 iMetaD calculations were performed.^55^ We emphasize that this HLDA CV is different from the LDA CV used in Section 3.2, and it was the CV that worked the best in ref (35). The rate constants and γ estimates are given in Figure 6. We found that fitting the CDFs for KTR and EATR both dramatically improve rate estimates as compared to the iMetaD estimator (performing at least comparably to the short time fitting in ref (35).). Moreover, in this approach, we are able to extract a γ parameter showing that, for chignolin, the RMSD to the native structure is a worse CV for predicting rates of unfolding than radius of gyration, in contrast to our Go-model example. As expected, based on the more accurate iMetaD results, γ for HLDA is much closer to 1, validating that it is a good CV for this problem. Although this CV already gives fairly good rate constant predictions for slow deposition, accounting for the fitted γ when using HLDA in both EATR and KTR results in nearly perfect rate predictions at all deposition times.

## 4 Conclusions

In this work, we have developed a general rate theory for time-dependent biased simulations that encompasses several of the existing methods by using the scaling factor *f*(*t*) (from eq 11) with different analytical formulations. In practice, LM and CDF-fitting are two different manners for estimating the unbiased rate constant from an ensemble of simulations launched from a single state. Although we demonstrate in this work that the previously proposed KTR approach works robustly for realistic problems, it does not predict as accurate results as iMetaD for the case of an ideal 1D CV. Therefore, we proposed the EATR formulation, which exactly coincides with iMetaD in the ideal CV case but which gives good rate estimates for bad coordinates more robustly than does iMetaD, with the additional benefit of reporting on the efficiency of that parameter through the parameter γ. Having a high value of γ seems to suggest that KTR or EATR will provide high quality rate constant predictions. We also foresee using γ as a metric that can help us in iteratively optimizing our choice of CV(s) for rate calculations. We do note though that having a good CV for a rate calculation may not be the same as having a good CV for predicting a full free energy surface, as it may only be a good way of describing the starting state and configurations on the way to a transition state.

We have validated the methods over the more complex landscape of protein G and chignolin unfolding where we could still have ground truth. We have found that both the KTR and EATR methods offer accurate rate measurements from biased simulations. The accuracy of the rates determined from these methods are surprisingly insensitive to biasing rate and CV quality, even for frequent-biasing regimes where the average maximum bias likely exceeds the true free energy barrier. We find that the γ computed from both KTR and EATR report CV efficiencies γ that correlate with our qualitative intuition of what are good or bad biasing coordinates. Overall, we find that the CDF fit is better than LM to obtain the rate constant. However, many solution pairs of {*k*_0_, γ} could pass the KS test, so getting a good fit is not sufficient to guarantee that the optimal unbiased rate constant was obtained; in practice the predicted values were all very close to the highest confidence prediction (as shown in Figure S6). Indeed, for cases where the biasing potential is well approximated as time-independent (such as OPES flooding), we find that optimizing both *k*_0_ and γ simultaneously brings unstable solutions. Nonetheless, by analyzing the unfolding rate constant as a function of a rate scaling function for different OPES barrier parameter values, we are able to overcome this, obtaining stable and accurate estimates.

We find that for biasing 2D landscapes, all methods perform reasonably well, which is an indication that biasing many directions might be helpful for barrier-crossing enhancement. In the future, we hope to test the methods on more complex systems using multiple biasing dimensions and, if necessary, extend the theory to multiple dimensions as was done for force-spectroscopy in refs (56 and 57).

Despite this success, there are several aspects of the EATR method that can still be improved through future work. For example, we could take into account the effect of the bias on the pre-exponential factor. Although the method works well in the case of our coarse-grained model of protein G for very fast biasing, we have not solved the general problem of how to compute rates in the overbiasing regime where γ times the bias could still be larger than the true barrier, as for example for LDA at fast deposition times (Figure 4), which could lead to the overestimated rate constants and small γ (Figure 3). Addressing this issue will be crucial for systematically using the EATR method for large systems with many slow degrees of freedom. It might be that using a slightly more time-consuming procedure like the OPES EATR-slope fit with multiple barrier heights is the best solution. Going forward, we would like to determine whether it is possible to find a theoretical interpretation for γ in multiple dimensions, e.g., whether it can be derived considering projection operator approaches, and investigate whether it is rigorously connected to nonequilibrium estimators of the effect of a time-varying bias on the rates.^54^

## 5 Computational Methods

### 5.1 Overdamped Langevin Dynamics on a 1D Potential

We ran overdamped Langevin dynamics over the potential given in eq 23 using *x*_0_ = −3, *x*_1_ = 3, and Δ*U* = 8 *k*_B_*T*. We used an integration time step of d*t* = 0.01 τ where τ is the time unit. The friction parameter used was 0.02 τ, which corresponds to the friction coefficient ζ = 50 τ^–1^ and the diffusion coefficient *D* = 0.02 τ*k*_B_*T*. All simulations were started from *x* = −3. Because the diffusion coefficient and potential are known, we can derive the standard Kramer’s expression in the Smoluchowski limit^38^ from eq 24
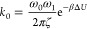
24and use that to determine the unbiased rate constant. For this specific system, the theoretical rate constant is 9.49 × 10^–7^ τ^–1^. These Langevin dynamics simulations were performed using the PESMD tool in PLUMED.^53^ Some of these simulations were biased using WT-MetaD with a starting hill height of 1 *k*_B_*T*, a σ of 0.5, and a biasfactor of 2.0. MetaD simulations were performed for four bias-deposition times (Δd*t*): 10^1^ τ, 10^2^ τ, 10^3^ τ, and 10^4^ τ.

### 5.2 Well-Tempered Metadynamics

In WT-MetaD simulations, a history-dependent biasing potential *V*(ξ, *t*) is generated at a position ξ in CV-space. *V*(ξ, *t*) is formed as a sum of Gaussians with width σ (which can differ for each CV) and height *h* deposited every Δ steps. For a one-dimensional CV, this can be written as
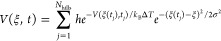
25where *t*_*j*_ = *j*Δd*t* are the times where hills were deposited prior to time *t*, and *N*_hills_ = ⌊*t*/(Δd*t*)⌋. Here, Δ*T* is a tempering factor which causes the heights to decrease proportionally to how much bias is already applied at that point, and is specified in PLUMED by setting a biasfactor of the form λ = (*T* + Δ*T*)/*T*, where *T* is the simulation temperature. In the original untempered MetaD, the hills are of constant height, i.e., Δ*T* → ∞. In iMetaD, the pace Δd*t* would be taken to be large, such that the frequency of deposition (Δd*t*)^−1^ becomes small. For notation simplicity, we have omitted the explicit dependence on ξ from eq 25 in all equations in the Theory.

### 5.3 On-The-Fly Probability Enhanced Sampling (OPES)

A history dependent biasing potential *V*(ξ, *t*) is used in OPES simulations, which is obtained from an estimate of the probability distribution made “on the fly” using kernel density estimation. *V*(ξ, *t*) is given by^30^
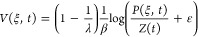
26where λ is the biasfactor from WT-MetaD, *P*(ξ, *t*) is the probability distribution estimate at time *t*, *Z*(*t*) is the normalization factor for *P*(ξ, *t*), and ε = e^–βΔ*E*/(1–1/λ)^, where Δ*E* is the barrier parameter. ε serves to prevent the bias from surpassing Δ*E*.

### 5.4 Go̅-like Model of Protein G

A Go̅-like coarse grained model of the B1 domain of protein G was prepared to assess the accuracy of the rate extraction methods, starting from PDB ID 1PGB. This system was selected because it was previously used as a paradigmatic example of a two state folder with known good and bad reaction coordinates.^48,58,59^ In a Go̅-like model, each residue is modeled as a bead at the position of the α-carbon. The force field for this model treats pseudobonds and angles harmonically, and pseudodihedrals using a Fourier series. Noncovalent interactions, as in ref (60), depend on whether the residues are in contact in the native structure, which is determined by whether the side chains of two residues contain heavy atoms within 4.5 Å of each other. The force field parameters for the model in refs (48 and 59) were provided by the authors. Our implementation of the potential in LAMMPS^61^ and input files for all simulations are provided in the GitHub for this article (see Data Availability below).

#### 5.4.1 Molecular Dynamics Simulations

The MD simulations of the Go̅-like model were performed using the LAMMPS software package.^61^ The software was updated partway through the project and the version used for each set of simulations is shown in Table S1. All simulations used a time step of d*t* = 10 fs, and the temperature was held constant at 312 K using the Nosé–Hoover chain thermostat^62^ with a damping factor of 1 ps and a chain length of 3. All simulations started from the folded structure.

For the unbiased simulations, we ran 200 replicates and ended the simulations when the protein model unfolded, which was defined to be when the CV *Q* decreased past 0.35 as described above. The empirical CDF for the transition times to the unfolded state were fit to eq 1 as explained in the Theory section to obtain the observed unbiased rate for this system.

#### 5.4.2 Collective Variables

A variety of CVs were analyzed for the Go̅-like model, which were used for the biased simulations and during the rate analysis. The first of these is the fraction of native contacts (*Q*), which captures the degree to which the protein is folded. This CV is the fraction of the contacts present in the native structure which are still present, and was defined as in ref (59). The end-to-end distance (*R*_ee_) was also used, as it was previously determined to be a poor coordinate.^48^ The RMSD of the protein with respect to the native structure and the radius of gyration (*R*_g_) were included to compare with the previously used CVs for this system.

To define the LD1 coordinate, first we performed a cluster scan on the unbiased trajectory using the shapeGMM clustering algorithm^63^ with 50,000 frames for training, 3 training sets and 15 attempts each, for cluster sizes (*K*) = 2,...,6. The training curve with cross validation from the scan is shown in Figure S2. We used the positions of the beads as input features for shapeGMM. We did a 5 state shapeGMM fit on the entire trajectory (∼1.2 M frames) with 15 attempts to identify the distinct clusters. We then performed an iterative global alignment of the trajectory to the global mean and covariance. Multistate Linear Discriminant Analysis (LDA) was performed on the globally aligned trajectory with frames from all 5 clusters. Only the first coordinate (LD1) out of four resulting LD coordinates has been used in this study.^49^ In Figure S2b,c we show that this coordinate completely separates the folded and unfolded states, with the other states appearing as intermediates.

#### 5.4.3 Biased Simulations

The collective variables and biasing for protein G were handled using PLUMED.^53^ The version of PLUMED used for each set of simulations is shown in Table S1. As is the case for the 1D potential, WT-MetaD was used to bias the simulations. A set of untempered MetaD simulations were also performed, the results of which are provided in the Supporting Information. The parameters used for the WT- and untempered MetaD simulations are given in Table S2. The values of σ were chosen for WT-MetaD according to the standard deviation of the biased CV in the folded state, and for untempered MetaD σ was chosen to be less than that used in WT-MetaD. We performed simulations at eight different bias deposition times (Δd*t*): 1, 10, 100, 200, 500 ps, 1, 5, and 10 ns. 100 simulations were performed for each Δd*t*. The simulations were halted when the protein was determined to have unfolded, or when either the wall-clock time reached 48 h or a total simulation time of 10 μs was reached.

We also performed 6 sets of 100 OPES simulations each for *Q* and *R*_ee_. These sets were run with Δ*E* values of 5, 7, 9, 11, 13, and 15 kJ/mol. We used the values of σ from the WT-MetaD simulations as the width of the kernels and used a kernel update time of 1 ns. We excluded bias in the region *Q* < 0.65 when biasing *Q* and the region *R*_ee_ > 2.9 when biasing *R*_ee_.

#### 5.4.4 Potential of Mean Force and Committor Analysis

A long simulation of protein G was performed and the potentials of mean force (PMFs) along various CVs were determined from the unbiased simulation data using
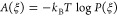
27where ξ is the CV along which the potential of mean force is computed and *P*(ξ) is the probability density of ξ obtained by computing a normalized histogram.

Committor analysis^33,51,52^ along *Q* was done on this long simulation by assigning either 0 or 1 to each frame of the trajectory depending on whether the system visits the folded or unfolded state next, then taking the average for all frames associated with each value of *Q*. In order to prevent incorrect assignments to either state, for the committor analysis the system was considered to be in the unfolded state when *Q* < 0.25 and to be in the unfolded state when *Q* > 0.85. From this, *Q* = 0.35 was decided to be the critical value for unfolding, as illustrated in Figure 2. This was chosen to be less than the transition state to prevent counting cases where the system enters the transition region, but fails to unfold.

### 5.5 Bootstrap Analysis

Errors were obtained from bootstrap analysis.^47^ For this analysis, a new set of transition times was constructed by choosing random simulations from the original set with replacement. Once the new set had the same size as the original set, the rate calculation was performed on the new set. This was repeated 100 times and the standard deviation of the log of the rate and γ across these new sets is reported.

### 5.6 Kolmogorov–Smirnov Test

The KS test was performed to assess whether the transition distribution is accurately described by the expected theoretical distribution. For the case of unbiased or iMetaD, it is a Poisson distribution. The 2-sample KS test was used for the unbiased and iMetaD analyses. This version of the test determines the maximum deviation of the observed CDF from two samples and gives a *p*-value which, when sufficiently low, allows us to conclude that the samples most likely did not come from the same underlying distribution. We consider the empirical and theoretical distributions to coincide if *p* > 0.05. The 1-sample KS test was used for the KTR and EATR analyses, as generating large random samples from their distributions took a significant amount of time. This version of the test determines the maximum deviation of the observed CDF for one sample from a theoretical CDF and gave the same results as the 2-sample test in all the cases that were checked.

## Data Availability

Inputs for simulations, LAMMPS code for the Go̅-like model, code for analysis, data for each system consisting of the value of the CVs and bias versus time, a script for clustering and generating the LDA coordinate, and code for generating figures are available at https://github.com/hocky-research-group/EATR-paper-2024.

## Supporting Information Available

Supplemental derivations, details of the clustering and position LDA analysis, several figures giving additional analysis of our rates methods, potentials of mean force along different CVs, results for biasing two CVs simultaneously, and a detailed list of simulation parameters used to produce the MD results (PDF)

## Footnotes

⊥ By convention, the term “rate” is often used in the Kramers’ problem when referring to the rate constant/coefficient, and so we also use the term rate when not specifically referring to the rate constant, *k*.^39^
