# Supplementary material for: Good Rates From Bad Coordinates: The Exponential Average Time-dependent Rate Approach
Source: J Chem Theory Comput. 2024 Jul 2;20(14):5901–12. doi: 10.1021/acs.jctc.4c00425 (PMC11270837; doi:10.1021/acs.jctc.4c00425)
Supplement: Supplementary file 1 — ct4c00425_si_001.pdf [file ct4c00425_si_001.pdf]

Supporting Information for: Good rates from bad coordinates: the exponential average time-dependent rate approach

Nicodemo Mazzaferro,[†] Subarna Sasmal,[†] Pilar Cossio,[*,‡,¶] and Glen M. Hocky[*,†,§]

†Department of Chemistry, New York University, NY, 10003, USA
‡Center for Computational Mathematics, Flatiron Institute, New York, 10010, USA
¶Center for Computational Biology, Flatiron Institute, New York, 10010, USA
§Simons Center for Computational Physical Chemistry, New York University, NY, 10003, USA

E-mail: pcossio@flatironinstitute.org; hockyg@nyu.edu

S1 iMetaD from the general time-dependent rate theory

Let simulation i have a history-dependent bias $V_i(t)$ which scales the rate for that simulation as

$$f_i(t) = e^{\beta V_i(t)} \,, \tag{S1}$$

where the "rate for a simulation" is the inverse of the mean observed transition time we would expect for a set of simulations experiencing the same bias potential. Substituting this into Eqs. 7 and 11 yields

$$S_i(t_i) = e^{-k_0 \int_0^{t_i} e^{\beta V_i(t')}\, dt'} \,, \tag{S2}$$

with

$$k_0^* = \frac{M}{\sum_{i=1}^{N} \int_0^{t_i} e^{\beta V_i(t')}\, dt'} \,. \tag{S3}$$

If all simulations transition and we define the acceleration factor α_i to be

$$\alpha_i = \frac{1}{t_i} \int_0^{t_i} e^{\beta V_i(t')}\, dt' \,, \tag{S4}$$

we can reinterpret the scaling of the rate constant as a scaling of time by α_i

$$S(t_i) = e^{-k_0 \alpha_i t_i} = e^{-k_0 t_i^{\text{rescaled}}} \,, \tag{S5}$$

with

$$k_0^* = \frac{N}{\sum_{i=1}^{N} \alpha_i t_i} = \frac{1}{\left\langle t_i^{\text{rescaled}} \right\rangle} \,, \tag{S6}$$

which are the survival function and rate estimator for iMetaD, respectively.

S2 Adding γ directly to the iMetaD rate scaling function

Consider if we had simply added γ to Eq. S1 to form a new rate theory. This would yield the rate scaling function

$$f_i(t) = e^{\beta \gamma V_i(t)} \,, \tag{S7}$$

which, when substituted into Eq. 11, gives the LM estimate for the rate constant

$$k_0^*(\gamma) = \frac{M}{\sum_{i=1}^{N} \int_0^{t_i} e^{\beta \gamma V_i(t')}\, dt'} \,, \tag{S8}$$

which can be substituted along with Eq. S7 into Eq. 10 to get a likelihood function which could in principle be maximized. However, if we do this and take the derivative with respect to γ, we find

$$\frac{d(\log \mathscr{L})}{d\gamma} = \sum_{i=1}^{M} \beta V_i(t_i) - k_0 \sum_{i=1}^{N} \int_0^{t_i} \beta V_i(t') e^{\beta \gamma V_i(t')} dt'. \tag{S9}$$

The first term is guaranteed to be zero if no bias is deposited at the barrier, since the system is at or past the barrier when it transitions at time t_i, and the second term is strictly less than zero. Therefore, there is no maximum in the likelihood function, so no LM estimate for the rate exists.

S3 Results for a matched harmonic potential where γ is fit rather than being restricted to $\gamma = 1$

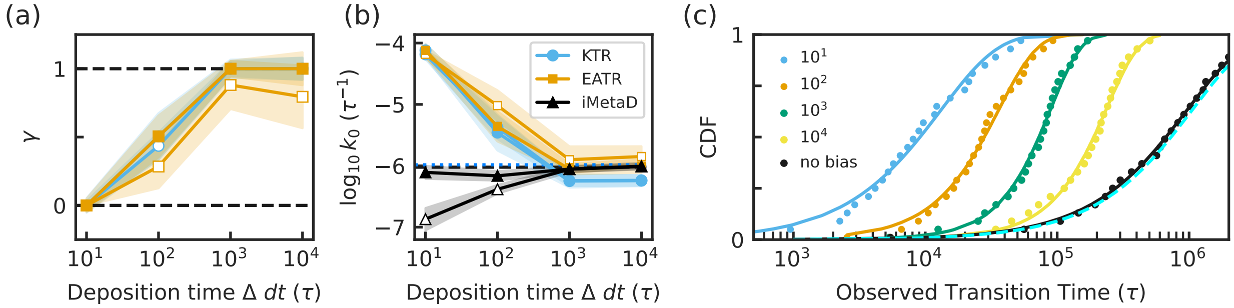

Figure S1: (a) γ obtained from the KTR and EATR methods, where the maximum likelihood estimates are represented by open symbols and the least-squares CDF fits are represented by filled symbols. (b) The rate constants obtained from the iMetaD, KTR, and EATR methods. iMetaD provides better rate estimates in this case because this is a perfect CV, which iMetaD assumes while KTR and EATR do not. (c) The best-fit EATR CDFs for each value of deposition time, $\Delta\, dt$.

S4 ShapeGMM and Position-LDA clustering analysis

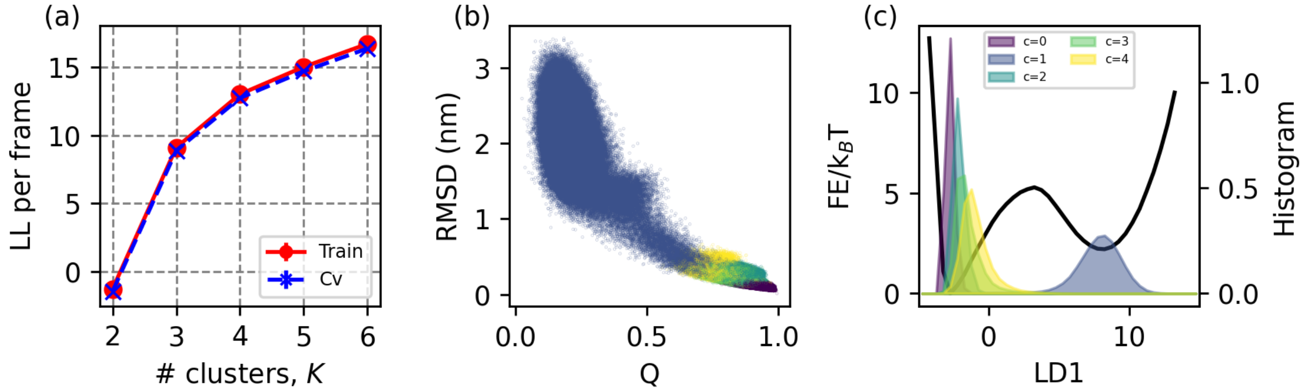

Figure S2: (a) Log-likelihoods of data coming from a shapeGMM model trained on K clusters as described in Sec. 5.4.2. The agreement between training and cross-validation prediction suggests that the data is not over-fit for any of these numbers of clusters. (b) 2D scatter plot of sampled conformations colored according to their cluster assignments from ShapeGMM using a $K = 5$ model. Based on the position of each cluster in (Q,RMSD) space, we assign the 0 to be the folded state and 1 the unfolded state (colors defined in c). (c) 1D PMF profile (solid black) along the LD1 coordinate. Histograms of LD1 corresponding to separate clusters, normalized individually and colored according to cluster id are shown in transparent colors.

S5 PMFs calculated for Gō-like model of Protein G from an unbiased trajectory

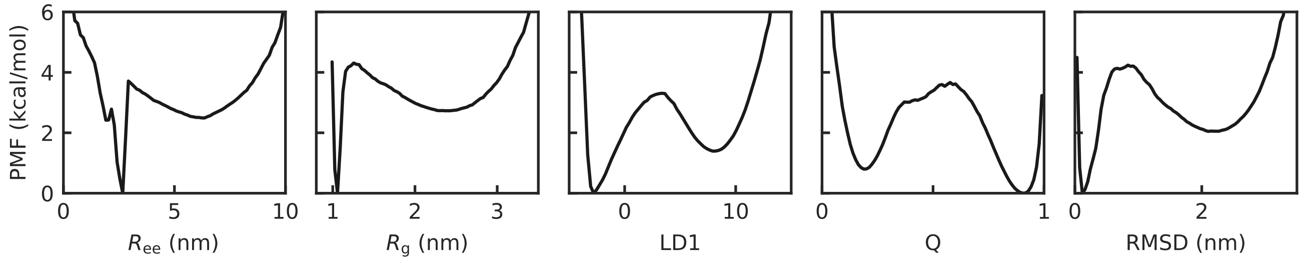

Figure S3: PMFs of the Gō-like model of Protein G computed along each of the five CVs presented in the Computational Methods section.

S6 Rate estimates using untempered MetaD

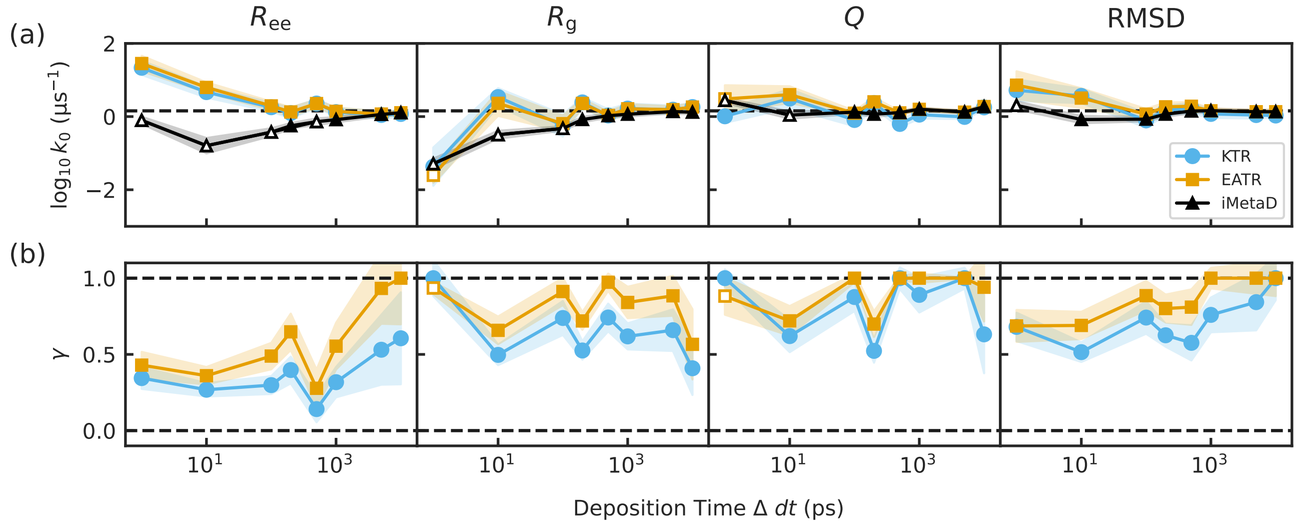

Figure S4: (a) The rate constants obtained from the four CVs biased using untempered MetaD using the iMetaD, KTR, and EATR methods. These methods provide accurate results in this biasing scheme. (b) The values of γ obtained from the KTR and EATR methods for each of the CVs.

S7 Distribution of bias deposited during MetaD

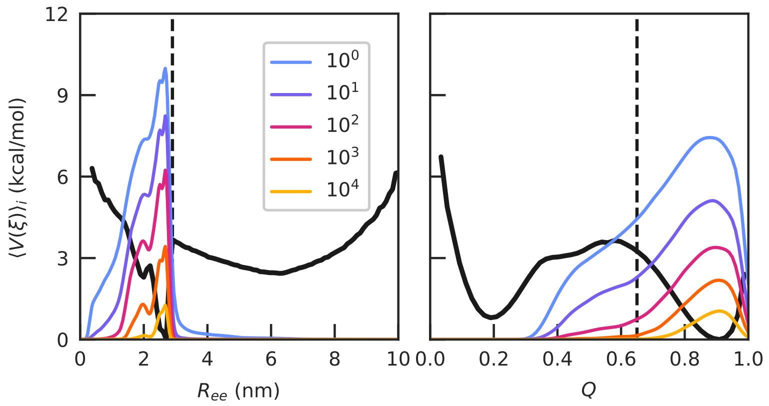

Figure S5: The averaged bias as a function of R_{ee} and Q for well-tempered MetaD simulations of protein G unfolding, for different values of $\Delta\, dt$ (in ps) shown in the legend. The vertical dashed lines represent the values of these CVs which were used for the excluded region in OPES fooding. The simulations with smaller $\Delta\, dt$ show overbiasing.

S8 Multiple k_0 and γ pairs pass the KS test

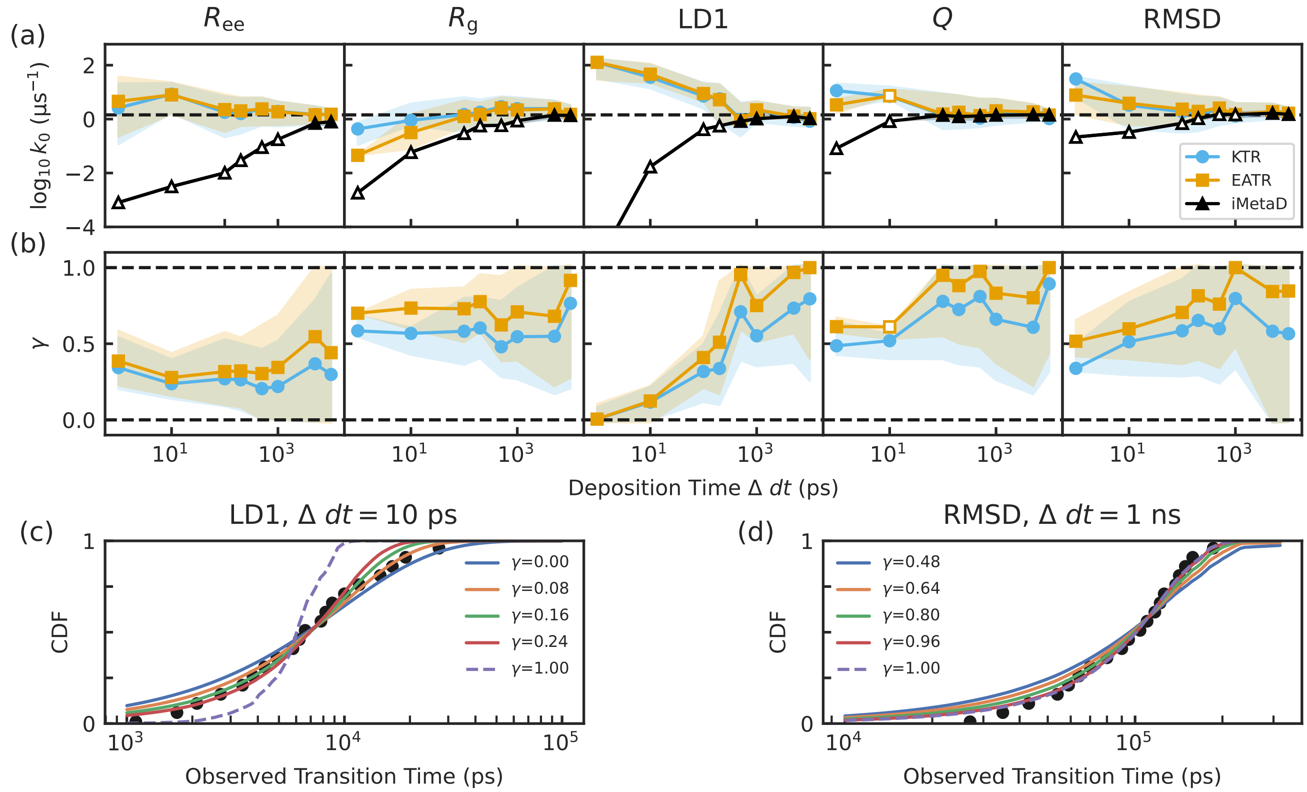

Figure S6: Same data as Fig. 3. (a) Shaded regions represent the ranges of rate constants for each of the five CVs from the iMetaD, KTR, and EATR methods which pass the KS test for some value of γ. (b) Shaded regions represent the ranges of γ values from the KTR and EATR methods which pass the KS test for some value of the rate constant. (c) Several EATR CDFs which pass the KS test for the LD1 CV at $\Delta\, dt = 10$ ps. (d) Several EATR CDFs which pass the KS test for the RMSD CV at $\Delta\, dt = 1$ ns.

S9 Rate estimation for OPES flooding

S9.1 k_0 and γ simultaneous optimization fails for constant bias

Because OPES produces a rough estimation of the free energy very quickly, the biasing potential averaged over many trajectories is effectively time-independent. While this is excellent for enhanced sampling applications, the KTR and EATR methods' k_0 and γ simultaneous optimization fails in cases where the rate is not time-dependent. To see why, consider what would happen if the bias $V_i(t) = V(\xi(t))$ were a constant function of the CV. Then, according to KTR, the biased observed rate constant (denoted k_{obs}) can be expressed as

$$k_{\text{obs}} = k_0 e^{\beta\gamma V_{\max}} , \tag{S10}$$

where the survival function is

$$S(t) = e^{-k_0 e^{\beta\gamma V_{\max}} t} . \tag{S11}$$

Clearly, we cannot fit both k_0 and γ, as they ultimately have the same effect on the survival function: changing the decay constant. Attempting to fit both, even for EATR (where $\left\langle e^{\beta\gamma V(\xi)} \right\rangle$ is constant instead), often causes unstable solutions in the optimization, e.g. shown in Fig. 5 where γ tends to 0 or 1. This leads to poor rate estimates.

S9.2 OPES-slope fit

We can overcome the unstable optimization solutions by using the barrier parameter in OPES to determine the relationship between the amount of bias and the biased observed rate constant in a procedure we call OPES-slope.

To do this, we ran sets of OPES simulations with different values for the barrier parameter. We measured the observed rate for each set of simulations as the inverse of the biased mean residence time for the simulations. We assume that the observed rate constant is given by

$$k_{\mathrm{obs}} = k_0 e^{\gamma g(\{V_i(t)\})} , \tag{S12}$$

where $\{\cdot\}$ denotes all values in a set of simulations, and the bias measure $g(\{V_i(t)\})$ is given by the rate scaling functions for KTR and EATR (Eqs. 16 and 19, respectively). For KTR $g(\{V_i(t)\}) = \beta \langle V_{\mathrm{max}} \rangle$, and for EATR we approximate $g(\{V_i(t)\}) \approx \log \left\langle e^{\beta V_i(t)} \right\rangle$, where $\langle \cdot \rangle$ is an average over the simulations in a set . Although plugging the EATR bias measure into Eq. S10 does not exactly lead to Eq. 19, we found that this approximation gave good results. In OPES, these bias measures are effectively constant, but to obtain a single value we took the time average for each simulation set. Finally, taking the natural logarithm of Eq. S12, we fit the observed rates as a function of the bias measures, for the sets of simulations, to

$$\log k_{\mathrm{obs}} = \log k_0 + \gamma g(\{V_i(t)\}) , \tag{S13}$$

to obtain estimates for k_0 and γ. In Fig. S7, we show $\log(k_{\mathrm{obs}})$ as a function of $g(\{V_i(t)\})$ for OPES simulations with different barrier parameter values for protein G unfolding along R_{ee} and Q. The fit of these points with Eq. S13 results in an accurate estimate of the unbiased rate and γ. The values from fitting are: KTR-slope: $k_0 = 2.38\ \mu s^{-1}$ and $\gamma = 0.51$ for Q, $k_0 = 1.30\ \mu s^{-1}$ and $\gamma = 0.29$ for R_{ee}; EATR-slope: $k_0 = 1.44\ \mu s^{-1}$ and $\gamma = 0.87$ for Q, $k_0 = 1.34\ \mu s^{-1}$ and $\gamma = 0.37$ for R_{ee}. The estimates of the unbiased rate constants are within 5% of the true rate.

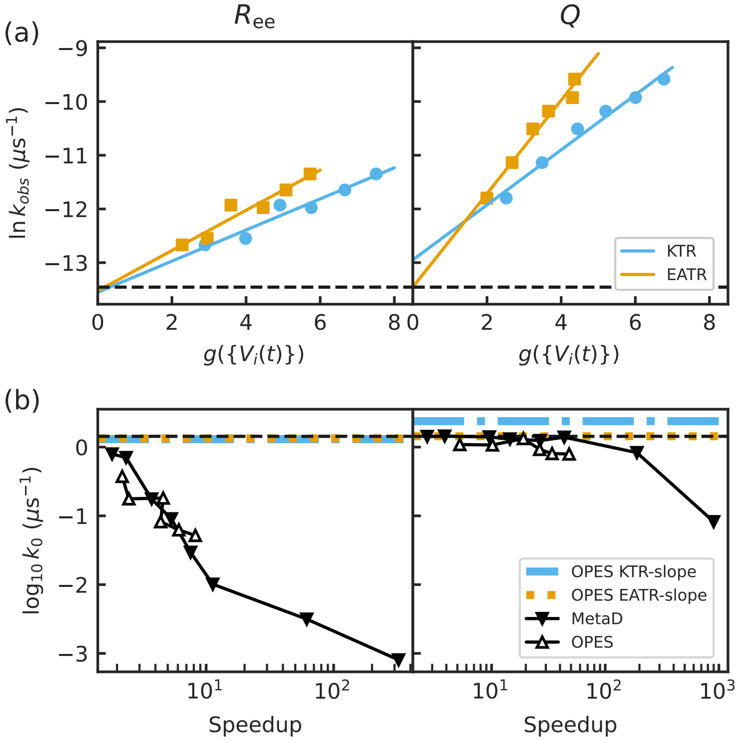

Figure S7: (a) The observed rate (inverse mean residence time in the biased simulation) for each set of simulations as a function of the bias measure $g(\{V_i(t)\})$ calculated for KTR and EATR. The black horizontal dashed line marks the true value of $\ln k_0$. (b) A comparison of the rate estimates for MetaD and OPES using the iMetaD estimator. The rates are plotted against "speedup", $\tau_{\mathrm{unbiased}}/\tau_{\mathrm{obs}}$ (see also Ref. 35). Both biasing schemes give similar results at any given amount of speedup. The blue and orange-dashed lines are the KTR and EATR rate estimates from the fit in panel (a).

S10 2D MetaD on R_{ee} and R_g

We performed MetaD simulations while simultaneously biasing the end-to-end distance R_{ee} and the radius of gyration R_g. For these two CVs, a combined PMF from the long unbiased trajectory is shown in Fig. S8a. The rate constants obtained from each method are shown in Fig. S8b, and all methods recovered the rate constant equally well, except for EATR at the fastest bias-deposition time, where the KS test failed. The corresponding CDFs and EATR fits are shown in Fig. S8c, confirming that the method is able to predict the distribution of transition times. For intermediate $\Delta\, dt \geq 10^2$ ps, the value for γ improved in this case over only biasing R_{ee} and improved slightly over only biasing R_g. This suggests that biasing multiple CVs simultaneously increases the efficiency of biasing without affecting the rate estimation. This might also be the reason why iMetaD preforms well in this case, while it failed for the same bias-deposition times when biasing individual CVs (see Fig. 3).

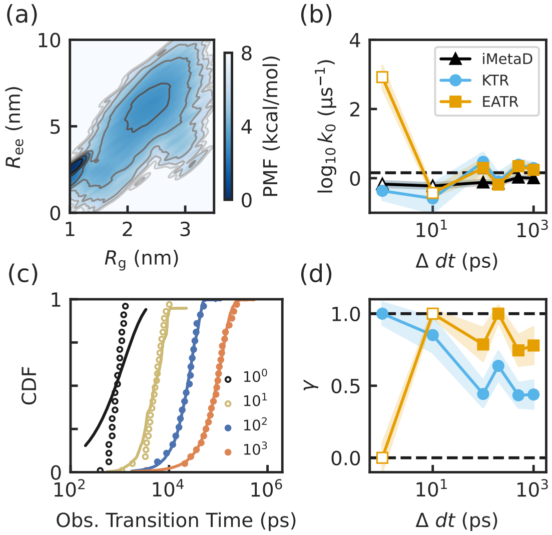

Figure S8: (a) The potential of mean force projected onto the 2D R_g/R_{ee} space. (b) The rate constants obtained from each method at various deposition times. The horizontal dashed line represents the true unbiased rate for this system. (c) The empirical and EATR-fit CDFs for each deposition time. The deposition times in the legend are given in ps. (d) The γ values obtained for KTR and EATR at various deposition times. In panels b, c, and d, open shapes indicate where the KS test failed. We performed some simulations with even slower biasing with the result that not every simulation transitioned; while the resulting rate constant estimate was unaffected, the estimated value of γ dropped significantly. The effects on the rate and γ are shown in SI Fig. S9.

S11 Effect of un-transitioned simulations on k_0 and γ

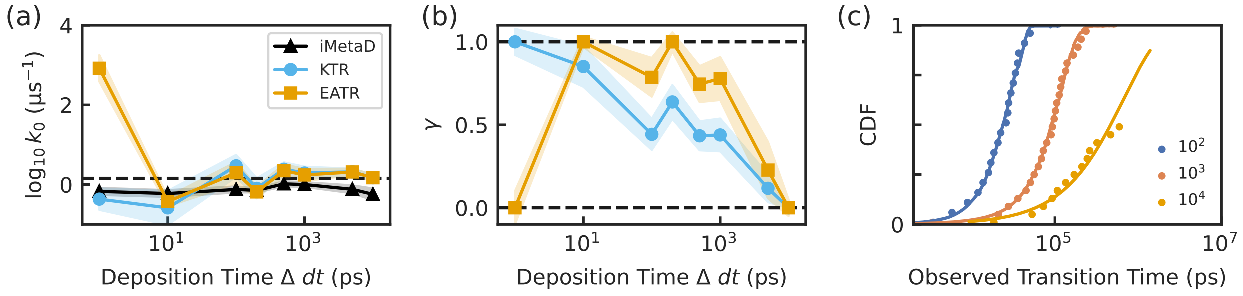

Figure S9: Same as Fig. S8, with additional slower deposition times where not all simulations transitioned. (a) The rate constants obtained from fitting the CDF for iMetaD, KTR, and EATR for the 2D R_{ee}/R_g CV. The rate constant estimates for $\Delta\, dt = 5$ ns and 10 ns are as accurate as the previous points, although only 66% and 50% of the simulations transitioned, respectively. (b) The values of γ obtained from KTR and EATR. The γ estimates for $\Delta\, dt = 5$ ns and 10 ns are significantly lower than the previous points. (c) The empirical CDF and the best fit EATR CDF for $\Delta\, dt = 100$ ps, 1 ns, and 10 ns. The KS test is not reported for incomplete simulation sets due to how the KS test is implemented in SciPy.

S12 Simulation software and parameters

Simulations were initially performed with the LAMMPS version from 5 May 2020 and PLUMED 2.6.6. We switched to a newer version of LAMMPS and PLUMED to perform simulations with the LDA coordinate, and due to an error in how the older version of PLUMED was computing Q, which we corrected in PLUMED 2.8*. The exact versions used for all of our simulations are given in Tab. S1.

Table S1: The version of each software used in each simulation set.

Simulations	LAMMPS	PLUMED
Unbiased 1D	—	v2.8.1
Biased 1D	—	v2.8.1
Unbiased GB1	5 May 2020	—
R_{ee}	5 May 2020	v2.6.6
R_g	5 May 2020	v2.6.6
LD1	23 Jun 2022	v2.8.3
RMSD	5 May 2020	v2.6.6
Q	23 Jun 2022	v2.8.3
R_{ee}/R_g (2D)	23 Jun 2022	v2.8.3
Q and R_{ee} OPES	23 Jun 2022	v2.8.3

Table S2: The MetaD parameters used for the well-tempered and untempered MetaD simulations for each CV.

Simulations	WT HEIGHT	WT SIGMA	WT BIASFACTOR	UT HEIGHT	UT SIGMA
1D	1.0 k_BT	0.5	2.0	—	—
R_{ee}	0.4 kJ/mol	0.06 nm	10.0	0.2 kJ/mol	0.01 nm
R_g	0.4 kJ/mol	0.02 nm	10.0	0.2 kJ/mol	0.01 nm
LD1	1.0 kJ/mol	0.5	6.0	—	—
RMSD	0.4 kJ/mol	0.02 nm	10.0	0.2 kJ/mol	0.01 nm
Q	0.6 kJ/mol	0.02	10.0	0.2 kJ/mol	0.01
R_{ee}/R_g (2D)	0.6 kJ/mol	0.06 nm/0.02 nm	10.0	—	—

*https://github.com/plumed/plumed2/pull/951
